# Prognostic ability of lung immune prognostic index in limited-stage small cell lung cancer

**DOI:** 10.1186/s12885-022-10351-7

**Published:** 2022-11-29

**Authors:** Bochen Sun, Qing Hou, Yu Liang, Shuqin Xue, Ningning Yao, Lijuan Wei, Xin Cao, Hongwei Li, Hongwei Si, Jianzhong Cao

**Affiliations:** 1 Department of Radiation Oncology, The Shanxi Province Cancer Hospital/Shanxi Hospital Affiliated to Cancer Hospital, Chinese Academy of Medical Sciences/Cancer Hospital Affiliated to Shanxi Medical University, No.3, Zhigongxin Street, Taiyuan, 030010 Shanxi China; 2 Department of Nuclear Medicine, the First Affiliated Hospital of Anhui Medical University, Hefei, 230022 Anhui Province China

**Keywords:** Lung immune prognostic index (LIPI), Limited-stage small-cell lung cancer (LS-SCLC), Prognostic biomarker, Immunity, Inflammation

## Abstract

**Background:**

Lung immune prognostic index (LIPI) is a prognostic marker of extensive-stage small cell lung cancer (ES-SCLC) patients received immunotherapy or chemotherapy. However, its ability in limited-stage SCLC (LS-SCLC) should be evaluated extensively.

**Methods:**

We retrospectively enrolled 497 patients diagnosed as LS-SCLC between 2015 and 2018, and clinical data included pretreatment lactate dehydrogenase (LDH), white blood cell count, and absolute neutrophil count levels were collected. According to the LIPI scores, the patients were stratified into low-risk (0 points) and high-risk (1–2 points). The correlations between LIPI and overall survival (OS) or progression-free survival (PFS) were analyzed by the Cox regression. Additionally, the propensity score matching (PSM) and inverse probability of treatment weight (IPTW) methods were used to reduce the selection and confounding bias. A nomogram was constructed using on multivariable Cox model.

**Results:**

Two hundred fifty and 247 patients were in the LIPI high-risk group and low-risk group, and their median OS was 14.67 months (95% CI: 12.30–16.85) and 20.53 months (95% CI: 17.67–23.39), respectively. In the statistical analysis, High-risk LIPI was significantly against worse OS (HR = 1.377, 95%CI:1.114–1.702) and poor PFS (HR = 1.338, 95%CI:1.1–1.626), and the result was similar after matching and compensating with the PSM or IPTW method. A novel nomogram based on LIPI has a decent level of predictive power.

**Conclusion:**

LIPI stratification was a significant factor against OS or PFS of LS-SCLC patients.

## Introduction

Lung cancer is the leading cause of cancer death worldwide [1, 2], and about 15% of the cases are small cell lung cancer (SCLC). Based on radiation fields, SCLC is usually stratified into the limited and the extensive stage (LS and ES) [3]. LS-SCLC were recommended to be concurrently treated with thoracic radiotherapy and 4–6 cycles of cisplatin + etoposide regimen [4]. However, their prognosis remains poor (median survival time: 16–24 months, 5-year survival rate: 25–33%) [5]. The confirmed prognostic factors of SCLC were gender, age, smoking, performance status (PS), and TNM staging system [6], but their ability was not so satisfactory. Therefore, It is very necessary to find a suitable clinical parameter for accurate risk stratification and personalized treatment.

Inflammation was profoundly related to the cancer prognosis, and some inflammatory or immune markers were developed for the patients [7]. Based on blood specimens, these biomarkers have low cost and reasonable practicability. For example, the lung immune prognostic index (LIPI) was calculated from the ratios of derived neutrophil-to-lymphocyte (dNLR) and lactate dehydrogenase (LDH) levels, and could subgroup patients into good, intermediate, and poor LIPI [8].

Recently, LIPI has been identified as a prognostic factor for NSCLC patients receiving targeted therapy, chemotherapy, and immunotherapy [8, 9]. In ES-SCLC, Qi et al. found that pretreatment LIPI score was correlated with the prognosis of first-line chemotherapy patients [10]. Li et al. analyzed 100 patients with ES-SCLC, they demonstrated that LIPI can also predict OS and PFS for patients treated with immunotherapy [11]. However, in LS-SCLC, due to only a small group of patients were enrolled (*n* = 77 and 66) [12, 13], the prognostic effect of LIPI was not observed in two known studies. Therefore, we designed this study to confirm the correlation between LIPI and the prognosis of LS-SCLC. Moreover, this study also aimed to develop a new nomogram to predict survival rates with high accuracy in LS-SCLC patients.

## Methods

### Patients

LS-SCLC patients were retrospectively enrolled at the Shanxi Cancer Center during June 2015 and February 2018. The criteria of eligible patients were: 1) pathologically confirmed SCLC; 2) in the limited-stage according to the Veterans Administration Lung Cancer Study Group (VALSG) staging system; 3) administrated chemoradiotherapy or chemotherapy; 4) could be followed including the results of laboratory and imaging examinations; 5) the initial timepoint was prior any anti-tumor treatment. The ethics committee at the Shanxi Province Cancer Hospital approved the protocol, and the review boards agreed to waive the requirement for informed consent.

### Data collection

Patient characteristics were retrieved from medical records, and laboratory data prior to treatment at least included LDH, white blood cell count (WBC), and absolute neutrophil count (ANC). According to the study from Mezquita et al. [8], LIPI was determined by dNLR (ANC/[WBC− ANC]) and LDH level, based on the following cutoffs: dNLR>3 and LDH> 260 U/L (our institution’s the upper limit of normal). Although the patients were often stratified into good (0 factors), intermediate (1 factor), and poor (2 factors) LIPI, in this study, only 22 cases (4.42%) were scored as poor LIPI. Therefore, the intermediate and poor LIPI were merged as the high risk group, and the remainder was as the low risk group (good LIPI). All patients underwent first-line platinum plus etoposide chemotherapy. The chemotherapy dose-adjustment (dose reductions or delay) was based on adverse effects. Early radiotherapy was started within the first 90 days of treatment or before the third cycle of chemotherapy [14, 15]. After radiotherapy and chemotherapy, PCI was performed in patients with complete response or partial response.

### Statistical analysis

Overall survival (OS) is defined as the time from diagnosis to death for any reason, and progression-free survival (PFS) is from the day of diagnosis to disease progression or death. Median PFS and OS were estimated by the Kaplan-Meier method, and survival curves were compared by the log-rank test. The variables with statistical significance were firstly identified by the univariate Cox regression (*P* < 0.05), and then were analyzed by the multivariate cox regression. Based on the results of multivariate Cox proportional hazards, a nomogram was constructed to predict 1-,2- and 3-year survival. The area under the receiver operating characteristic curve (ROC, AUC) was used to evaluate the quality of classifier.

To minimize the selection bias of our study, the nearest-neighbor matching (1:1) of the propensity score matching (PSM) method was used with a caliper distance of 0.02. Additionally, the inverse probability treatment weighting (IPTW) method was used to compensate for the missing not at random (MNAR) results from PSM. Sample balancing variables included age, gender, smoking history, TNM stage, chemotherapy cycle, RT, PCI, and treatment modalities. The chi-square test was used to compare the subgroups of patient characteristics. All data were analyzed by the R package (Version 3.6.3).

## Results

Among the enrolled patients (*n* = 497), the most individuals were male (80.7%), smoking (74.4%), younger than 60 years old (54.1%), the Eastern Cooperative Oncology Group (ECOG) score of 0–1 (83.7%), and in AJCC TNM 8th stage III (89.9%). They accepted chemotherapy alone (47.9%), early (23.1%), and late radiotherapy (29.0%). Furthermore, 12.7 and 68.8% of patients received prophylactic cranial irradiation (PCI) and more than four cycles of chemotherapy, respectively. Detailed patient characteristics are listed in Table 1.

**Table 1:** Clinical features of LS-SCLC patients according to LIPI status

Variables	Un-matched (*N* = 497)			After PSM (*N* = 398)			After IPTW (*N* = 993)		
	Low Risk (*n* = 250)	High Risk (*n* = 247)	*P* value	Low Risk (*n* = 199)	High Risk (n = 199)	*P* value	Low Risk (*n* = 497.3)	High Risk (*n* = 495.7)	*P* value
age60			0.813			0.688			0.882
< 60	116(46.4%)	112(45.3%)		95(47.7%)	91(45.7%)		229.0 (46.0%)	225.9 (45.6%)	
> =60	134(53.6%)	135(54.7%)		104(52.3%)	108(54.3%)		268.4 (54.0%)	269.8 (54.4%)	
gender			0.399			0.370			0.564
Female	52(20.8%)	44(17.8%)		41(20.6%)	34(17.1%)		99.4 (20.0%)	91.9 (18.5%)	
Male	198(79.2%)	203(82.2%)		158(79.4%)	165(82.9%)		398.0 (80.0%)	403.8 (81.5%)	
smoke			0.397			0.728			0.483
No	68(27.2%)	59(23.9%)		51(25.6%)	48(24.1%)		130.2 (26.2%)	120.2 (24.2%)	
Yes	182(72.8%)	188(76.1%)		148(74.4%)	151(75.9%)		367.1 (73.8%)	375.5 (75.8%)	
KPS			0.034			1.000			0.860
0–1	218(87.2%)	198(80.2%)		170(85.4%)	170(85.4%)		414.8 (83.4%)	411.3 (83.0%)	
2	32(12.8%)	49(19.8%)		29(14.6%)	29(14.6%)		82.6 (16.6%)	84.4 (17.0%)	
AJCC TNM 8th			0.001			1.000			0.887
I-II	36(14.4%)	14(5.7%)		12(6.0%)	12(6.0%)		49.9 (10.0%)	48.4 (9.8%)	
III	214(85.6%)	233(94.3%)		187(94.0%)	187(94.0%)		447.4 (90.0%)	447.3 (90.2%)	
cycles			0.020			1.000			0.947
< 4	66(26.4%)	89(36.0%)		59(29.7%)	59(29.7%)		153.8 (30.9%)	154.3 (31.1%)	
> =4	184(73.6%)	158(64.0%)		140(70.3%)	140(70.3%)		343.5 (69.1%)	341.5 (68.9%)	
RT			0.166			0.688			0.737
No	112(44.8%)	126(51.0%)		94(47.2%)	90(45.2%)		239.2 (48.1%)	233.1 (47.0%)	
Yes	138(55.2%)	121(49.0%)		105(52.8%)	109(54.8%)		258.2 (51.9%)	262.6 (53.0%)	
PCI			0.005			1.000			0.954
No	208(83.2%)	226(91.5%)		183(92.0%)	183(92.0%)		434.3 (87.3%)	433.5 (87.4%)	
Yes	42(16.8%)	21(8.5%)		16(8.0%)	16(8.0%)		63.0 (12.7%)	62.2 (12.6%)	
Treatment			0.303			0.897			0.905
early RT	64(25.6%)	51(20.7%)		46(23.1%)	46(23.1%)		113.5 (22.8%)	118.8 (24.0%)	
CT only	112(44.8%)	126(51.0%)		94(47.2%)	90(45.2%)		239.2 (48.1%)	233.1 (47.0%)	
late RT	74(29.6%)	70(28.3%)		59(29.7%)	63(31.7%)		144.6 (29.1%)	143.8 (29.0%)	

*LS-SCLC* limited-stage small cell lung cancer, *LIPI* lung immune prognostic index, *ECOG* The Eastern Cooperative Oncology Group performance status, *AJCC TNM 8th* the 8th edition of the TNM staging system, *CT* chemotherapy, *RT* radiotherapy, *PCI* prophylactic cerebral irradiation

There were 250 and 247 patients in the high-risk and low-risk groups, respectively. The characteristics of KPS, TNM stage, chemotherapy cycle, and PCI had significant differences between the LIPI subgroups. Using the PSM (*n* = 398) and the IPTW (*n* = 993) method, the factors of age, gender, smoking history, TNM stage, chemotherapy cycle, RT, PCI, and treatment modalities were controlled and well balanced (Table 1).

The median OS and PFS of the patients (*n* = 497) were 17.30 months (95%CI: 15.85–18.75) and 9.07 months (95%CI:8.30–9.84), respectively. In the univariate cox analysis, eight factors were significantly against OS and seven against PFS (Table 2). In the multivariate analysis, the factors of gender, chemotherapy cycles, thoracic irradiation, PCI, and LIPI were significantly against both OS and PFS.

**Table 2:** Univariate and multivariate analysis of the associations between LIPI and clinical outcomes of LS-SCLC

Variables	OS			PFS		
	Univariate	Multivariate		Univariate	Multivariate	
	*P*-value	HR (95%CI)	*P*-value	*P*-value	HR (95%CI)	*P*-value
Age	0.027			0.071		
Gender	0.001	0.620(0.466–0.825)	0.001	0.006	0.701(0.542–0.905)	0.007
Smoke	0.017			0.028		
ECOG	0.055			0.002	1.547(1.19–2.012)	0.001
AJCC TNM 8th	0.001			0.01		
CT cycles	< 0.001	2.023(1.61–2.542)	< 0.001	< 0.001	1.482(1.183–1.855)	0.001
RT	< 0.001	1.546(1.234–1.937)	< 0.001	< 0.001	1.296(1.046–1.606)	0.018
PCI	< 0.001	0.455(0.297–0.696)	< 0.001	< 0.001	0.548(0.394–0.764)	< 0.001
LIPI	< 0.001	1.377(1.114–1.702)	0.003	< 0.001	1.338(1.1–1.626)	0.004

*LS-SCLC* limited-stage small cell lung cancer, *LIPI* lung immune prognostic index, *ECOG* The Eastern Cooperative Oncology Group performance status, *AJCC TNM 8th* the 8th edition of the TNM staging system, *CT* chemotherapy, *RT* radiotherapy, *PCI* prophylactic cerebral irradiation, *OS* overall survival, *PFS* progression-free survival, *HR* hazard ratio, *CI* confidence interval

**Fig. 1:**
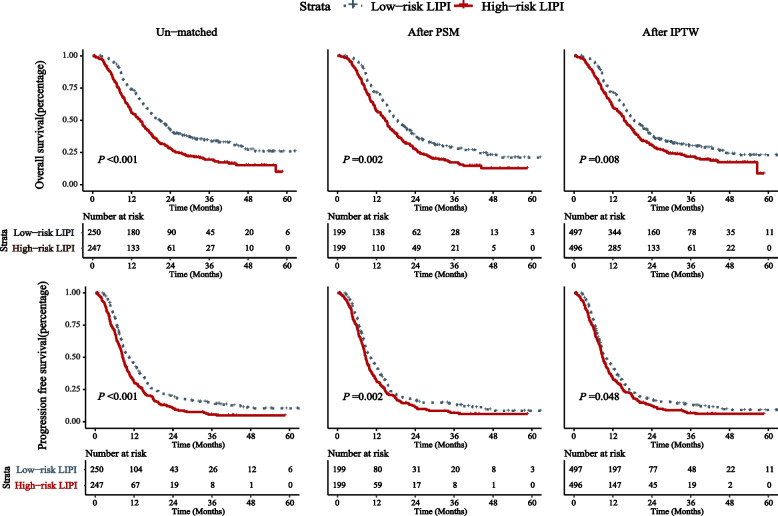
Survival plots for lung immune prognostic index (LIPI) status in limited-stage small cell lung cancer (LS-SCLC) patients

**Fig. 2:**
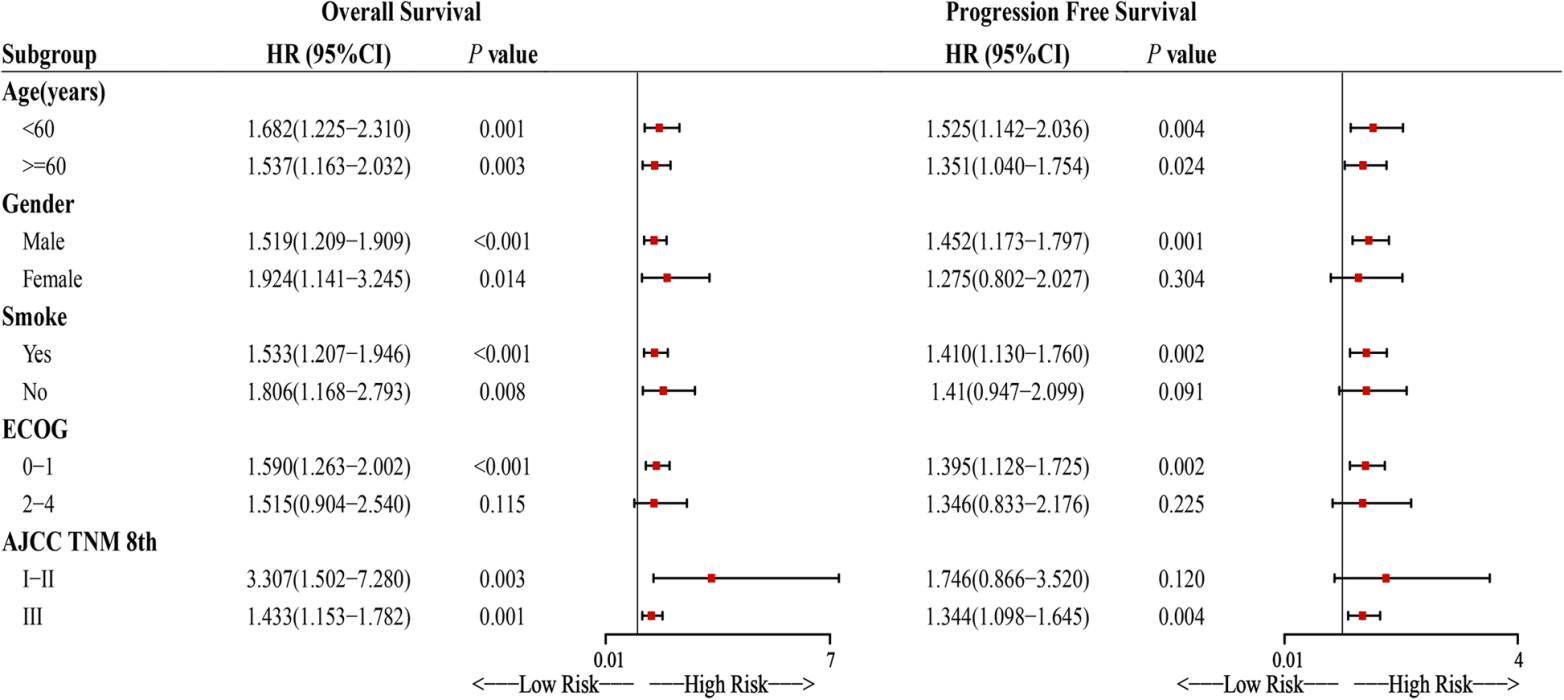
Subgroups analyses for overall survival and progression-free survival. Abbreviations: ECOG, The Eastern Cooperative Oncology Group performance status; AJCC TNM 8th, the 8th edition of the TNM staging system; HR, hazard ratio; CI, confidence interval

The high-risk LIPI is related to worse OS (HR: 1.377, 95% CI: 1.114–1.702) and PFS (HR: 1.338, 95% CI: 1.100–1.626). The median OS was 14.67 months (95% CI: 12.30–16.85) and 20.53 months (95% CI: 17.67–23.39) for the high- and low-risk groups, respectively. The 1- and 3-year OS rates in the high-risk group were 55.4 and 19.8%, and in the low-risk group were 73.4 and 31.4%, respectively (*P* < 0.001). The median PFS was 12.45 months (95% CI: 10.74–14.17) and 18.50 months (95% CI: 15.89–21.12) for the high- and low-risk groups, respectively. The high-risk group’s 1–3-year PFS rates were 30.7, 5.7, 44.6, 14.6% in the low-risk group, respectively (*P* < 0.001). After the PSM or IPTW adjustment, LIPI also significantly against either OS or PFS (Figs. 1 and 2).

In the stratified analysis, as shown in Fig. 2, the risk of progression in the high-risk group increases significantly among patients in males (HR = 1.452, 95%CI: 1.173–1.797) with a smoking history (HR = 1.410, 95%CI: 1.130–1.760), ECOG 0–1 (HR = 1.395, 95%CI: 1.128–1.725) and staged III (HR = 1.344, 95%CI: 1.098–1.645). Except ECOG, survival difference between the high-risk and low-risk groups existed in the subgroup analysis of others (Fig. 2).

**Fig. 3:**
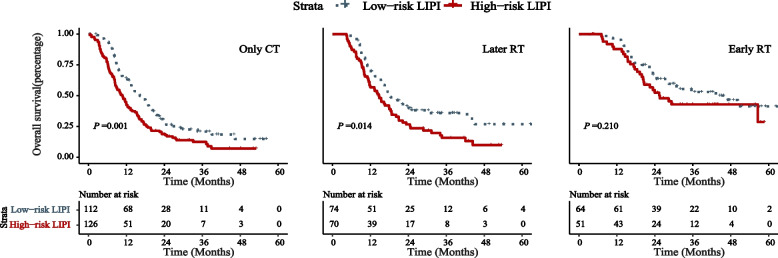
Overall survival plots for LIPI status according to therapy modality. Abbreviations: LIPI, lung immune prognostic index; CT, chemotherapy; RT, radiotherapy; Only CT: chemotherapy alone; Later RT: later chemoradiotherapy; Early RT: early chemoradiotherapy

Additionally, the influences of treatment modalities on the relationship between LIPI stratifications and survival were analyzed (Fig. 3). Compared to the high-risk LIPI group, a significant OS benefit was observed in the low-risk LIPI group for chemotherapy only (median: 16.23 vs. 10.70 months, *P* = 0.001) or late radiotherapy (18.33 vs. 14.67 months, *P* = 0.014). However, because of the intersection of the curves after 48 months, significant benefit for early radiotherapy did not exist (43.3 vs. 25.57 months, *P* = 0.210).

**Fig. 4:**
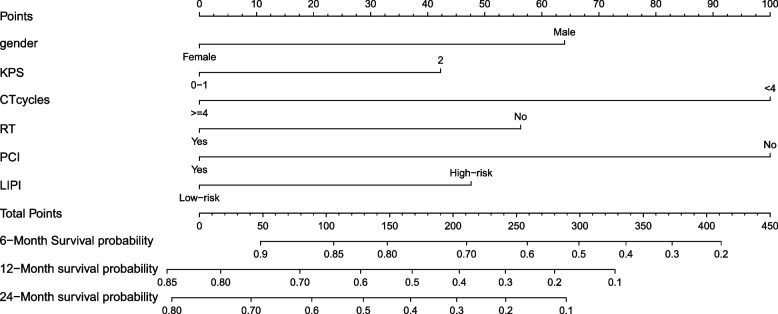
A nomogram for prediction of 1-, 2- and 3-year overall survival in limited stage small cell lung cancer. Abbreviations: ECOG PS, The Eastern Cooperative Oncology Group performance status; CT, chemotherapy; RT, radiotherapy; LIPI, lung immune prognostic index; PCI, prophylactic cerebral irradiation

**Fig. 5:**
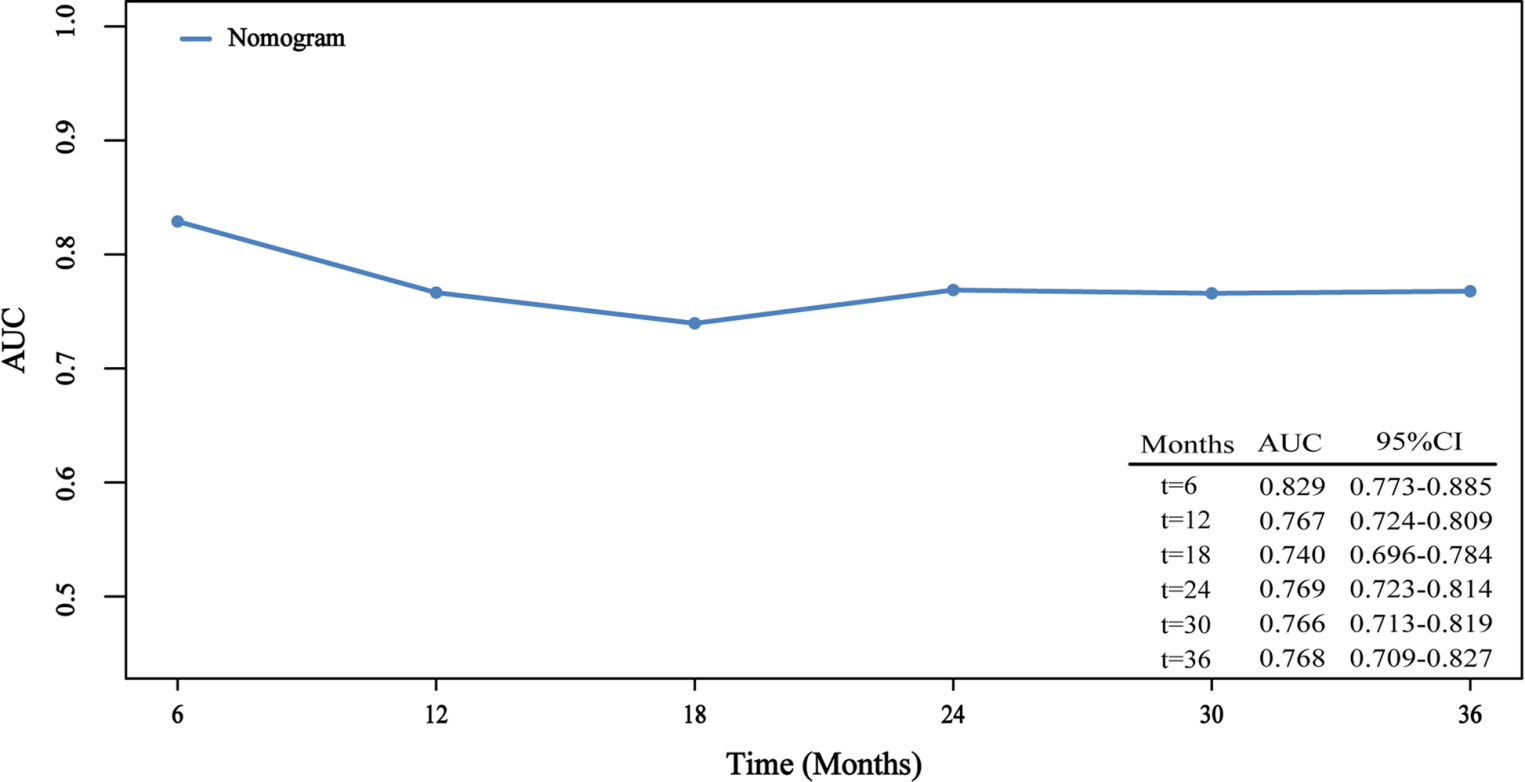
The time-dependent ROC curve of the nomogram. Abbreviations: ROC curve，receiver operating characteristic curve; AUC, Area Under Curve; CI, confidence interval

A nomogram was constructed based on the results of multivariate Cox proportional hazards. CT cycle and PCI have the greatest impact on prognosis, followed by gender, RT and LIPI (Fig. 4). The time-dependent ROC curve shows that the predictive power of the model is highest at 6 months. The model efficiency is stable, and the AUC value is stable at around 0.76 (Fig. 5).

## Discussion

Our results indicate that LIPI is an independent prognostic marker for both PFS and OS, and high-risk LIPI is associated with tumor progression and increased death rates.

Numerous studies have demonstrated that inflammatory cells can alter the tumor microenvironment (TME), and are involved in biological processes [16, 17]. For example, neutrophils are engaged and activated by cytokines and chemokines which regulate tumor growth, metastasis, and angiogenesis subsequently [18]. On the other hands, lymphocytes, especially tumor-infiltrating lymphocytes (TILs), play an anti-tumor role by regulating the immunologic function including cytotoxic cells [19]. Therefore, the elevated dNLR, as an index related to both neutrophilia and lymphopenia, can predict poor prognosis [10].

Additionally, as a critical hallmark of solid tumors, the glucose metabolism of malignant cells is reprogrammed to aerobic glycolysis (Warburg effect) [20]. In the last step, LDH is the enzyme responsible for the catabolism of pyruvate into lactic acid [21]. Therefore, LDH level is the marker of tumor activity [22], and is also confirmed as a prognostic marker of SCLC patients [23].

Above all, LIPI scored by dNLR and LDH might be a prognostic marker of NSCLC and ES-SCLC patients; however, because of the limited sample sizes of the published studies (*n* = 66 and 33) [12, 13], its ability in LS-SCLC needed to be confirmed by extensive sample researches. Additionally, the results of retrospective studies were easily deviated by bias; therefore, we not only collected a relatively large sample size (*n* = 497), but also used the PSM and IPTW methods to ensure the reliability of our results.

In this study, besides LIPI, other prognostic factors were also identified. Compared to males, females could benefit from chemotherapy, and had longer OS and PFS which coincided with the published studies [24]. Although ECOG was significantly against both OS and PFS in previous studies [25], because of the excellent performance status of LS patients, it did not relate to OS in ours. Additionally, our results indicated that the treatment-related factors of chemotherapy cycles, radiotherapy, and PCI were significantly correlated to the prognosis of LS-SCLC. As noted in a meta-analysis [26], after completing the prescribed chemotherapy regimen, earlier thoracic radiotherapy could improve OS of LS-SCLC patient. Therefore, clinicians should pay more attention to these treatment-related factors, especially chemotherapy compliance. Additionally, smoking is an inflammation-inducing behavior related to cancer development [27]. In our results, the PFS of high-risk LIPI was short in smoking males which could be explained by their prevalence in man.

We also found that different treatments affect the relationship between LIPI and survival. The results presented, unlike late RT patients, high-risk LIPI was not associated with worse prognosis in early RT patients. The mortality after 2 years decreased significantly in these patients, showing a long-term survival benefit. Similarly, this benefit also was observed in a study of stage III NSCLC [28]. In this study, LIPI significantly associated with 5-year survival for sequential treatments, but did not for concurrent chemoradiotherapy. In the concurrent treatments group receiving radiotherapy relatively early, LIPI survival curves intersected, and the prognosis of high-risk LIPI patients was even better after 36 months. Therefore, the outcome of patients might relate to the time point of radiotherapy intervention. It had been reported that tumor cells injured by chemoradiotherapy could promote the infiltration of anti-tumor immune cells (such as CD8+ T cells), and reshape the immune response of TME [29]. If radiotherapy was administered earlier, the immune cells could reach a higher level locally, and offset the long-term adverse prognosis of high-risk LIPI.

In addition, the analysis of different treatments reflects the limitations of LIPI. As an indicator of peripheral blood inflammation, LIPI does not reflect the local inflammatory state of tumor. Immune cells include peripheral blood and tumor-infiltrating cells which are interactively transformed and influenced. Peripheral blood immune cells correspond to the immune state of individuals and have the ability to activate and maintain immune response [7]. Tumor-infiltrating immune cells is associated with anti-tumor and immune evasion [30]. The two compositions were often studied separately; therefore, as indicated by these results, the combined analysis might reflect complete immune status and provide a more reliable basis for clinical decision.

Nomogram is a visualize risk scores for estimating tumor prognosis [31]. More and more nomograms are being developed for various cancers. Our nomogram involve clinicopathological features and blood markers, its included variables are comprehensive and the model show good accuracy. To our knowledge, this is the first nomogram based on LIPI to predict survival rate of lung cancer. Future external validation is needed to further verify its diagnostic potential.

There were some limitations in our study. First, although this study’s sample size is large, the number of patients with poor LIPI is small, which may not accurately assess the clinical characteristics and prognosis of patients with poor LIPI. Second, Our study included some patients with substandard treatment, such as insufficient chemotherapy cycles, late radiotherapy, or no PCI. However, in clinical practice, many patients do not receive standard treatment for aging, weakness, complications, and other reasons. Our results might help provide a reference for these patients. Third, PET-CT scan was not widely available, which may lead to clinical staging errors. Finally, This study was a single-center retrospective analysis, and selection bias was present due to the lack of external validation from another constitution. The prognostic ability of LIPI in LS-SCLC still needs to be further verified by a large sample, multicenter, and high-quality study.

## Conclusions

Our study indicates the prognostic ability of LIPI in limited-stage small-cell lung cancer patients. To get repeatable results from this single-center study, we used the PSM and IPTW methods that indicated that, either after matching or compensating the cases, LIPI stratification was a significant factor against OS or PFS of LS-SCLC patients.

## Data Availability

The datasets used and analyzed during the current study are available from the corresponding author on reasonable request.

## Abbreviations

LIPI: Lung immune prognostic index
ES-SCLC: Extensive-stage small cell lung cancer
LS-SCLC: Limited-stage SCLC
OS: Overall survival
PFS: Progression-free survival
PSM: The propensity score matching
IPTW: Inverse probability of treatment weight
PS: Performance status
ECOG: The Eastern Cooperative Oncology Group score
dNLR: Neutrophils/ (leukocytes minus neutrophils)
LDH: Lactate dehydrogenase
ICIs: Immune checkpoint inhibitors
VALSG: The Veterans Administration Lung Cancer Study Group staging system
WBC: White blood cell count
ANC: Absolute neutrophil count
MNAR: Missing not at random
PCI: Prophylactic cranial irradiation
TME: Tumor microenvironment
TILs: Tumor-infiltrating lymphocytes
ROC curve: Receiver operating characteristic curve
AUC: Area Under Curve
